# The Experience of Older People in the Shared Decision-Making Process in Advanced Kidney Care

**DOI:** 10.1155/2016/7859725

**Published:** 2016-11-20

**Authors:** Nicola Thomas, Karen Jenkins, Breeda McManus, Brian Gracey

**Affiliations:** ^1^London South Bank University, 103 Borough Road, London SE1 0AA, UK; ^2^Kent Kidney Care Centre, East Kent Hospitals University NHS Foundation Trust, Canterbury, UK; ^3^Barts Health NHS Trust, London, UK; ^4^Patient and Carer Group, Barts Health NHS Trust, London, UK

## Abstract

*Introduction*. This qualitative descriptive study was designed to understand the experiences of older people (>70 years) when making a decision about renal replacement therapy. This was a coproduced study, whereby patients and carers were involved in all aspects of the research process.* Methods*. A Patient and Carer Group undertook volunteer and research training. The group developed the interview questions and interviewed 29 people who had commenced dialysis or made a decision not to have dialysis. Interview data were transcribed and analysed, and common themes were identified.* Results*. 22 men and 7 women (mean age 77.4 yrs) from two hospitals were interviewed. 18 had chosen haemodialysis, 6 peritoneal dialysis, and 5 supportive care. The majority of patients were involved in the dialysis decision. Most were satisfied with the amount of information that they received, although some identified that the quality of the information could be improved, especially how daily living can be affected by dialysis.* Conclusion*. Our findings show that overall older patients were involved in the dialysis decision along with their families. Our approach is innovative because it is the first time that patients and carers have been involved in a coproduced study about shared decision-making.

## 1. Introduction

Shared decision-making (SDM) is the conversation that happens between a patient and their health professional to reach a healthcare choice together. This conversation needs patients and professionals to understand what is important to the other person when choosing a treatment [1]. The purpose of this study is to find out about the experiences of older people who have recently made a choice about having dialysis or not, in order to contribute to the evidence base for advanced kidney care practice.

It is possible that some people who have advanced kidney disease (AKD) may not wish to participate in decision-making; some may wish to share their decisions solely with their family; others may prefer to use a structured decision aid. In a systematic review of the factors that influence decision-making in adults living with kidney disease, Murray et al. [2] suggested in their conclusion that although patient decision aids and implementation of shared decision-making have been evaluated in patients with other medical conditions, little is known about interventions to support patients with AKD when making quality decisions.

It is hoped that the findings will inform the practice of members of local AKD teams, to provide a range of different decision-making models which are individualised according to patient preference.

## 2. Literature Review

Shared decision-making is a process by which “clinicians and patients work together to select tests, treatments, management, or support packages, based on clinical evidence and the patient's informed preferences” [3]. Shared decision-making (SDM) is important for patients because of potential benefits such as improved physiological parameters through self-management programmes [4] and increased patient satisfaction [5].

People with AKD have to make difficult and complex decisions about their future care including which type of dialysis may suit them best or whether to have dialysis at all. A recent systematic review into the needs of older people with advanced kidney disease [6] made recommendations for further study into this area, as their review revealed a lack of research regarding the education requirements of the older person with AKD who has been asked to make a decision regarding dialysis or supportive care.

Two systematic reviews in kidney care found that patients' participation in the decision-making process was influenced by relationships with others; possible disruption to current lifestyle; desired degree of control (such as whether to self-care or not); and personal views on the benefits and risks of the treatment [2, 7].

A National US Study of Public Preferences [8] found that older people tended to prefer a physician-directed style of care, independent of health status. A survey of patients in Sweden [9] also found that older patients were more likely to defer to physicians for decisions about treatment independent of the presence of chronic illness. Whilst a number of other studies have suggested this pattern, limitations in study design have confounded age-related decline in health with preferences for a physician-directed style [8].

There is anecdotal evidence that people of different ages and also those from different cultures and ethnic groups have differing approaches to SDM. However, there is little evidence to back up this assertion. In the systematic review of the factors that influence decision-making in adults living with kidney disease, Murray et al. [10] suggested in their conclusion that more research is required to enhance our understanding of how these factors vary across the course of the disease by culture. Some studies undertaken in other conditions and contexts which suggest that patient preferences in SDM may be based on cultural-ethnic beliefs. Various studies have identified a family-centred model of decision-making, in contrast with an informative or shared model of decision-making [11]. Tariman et al. [12] found a diverse group of factors that informed clinical decision in older people with cancer, including personal beliefs and values, ethnicity, and previous health-related experience.

This research is important as it is the first study to our knowledge that aims to understanding of the experience of older people who have recently had to make difficult choices about their long term renal therapy.

## 3. Aims/Objectives

The overall aim of this study was to explore, during face-to-face interviews, the experiences of older people (>70 years) in the shared decision-making process in advanced kidney care. The objectives were

- to convene a specific Patient and Carer Group (PCG) to colead the project;
- to train the PCG members on the research process and in addition to facilitate volunteer training for the group members as per each hospital's requirements;
- for members of the PCG to develop interview questions and to then undertake semistructured interviews with older people who have had to make a decision about dialysis care in the past six months;
- to transcribe and analyse the data using thematic data analysis;
- to evaluate the role of the PCG in the project.

Findings will indicate if adaptations of existing decision-making models or decision aids and/or available material are required for the older person. Learning from this project (e.g., key messages about patient and carer training needs) will inform future projects that will involve service users as coresearchers.

## 4. Methods

This was a qualitative descriptive study, carried out in two hospitals in the UK: a large inner-city teaching hospital with a diverse population and a smaller hospital with a mostly white population. Ethical approval was obtained from the national Integrated Research Application System (IRAS) in the UK and, in addition, the local ethics committee in each hospital. Data on age, gender, ethnicity, and type of therapy (haemodialysis, peritoneal dialysis, or supportive care) were recorded but anonymised. Transcripts of interviews were identified by code (not name).

### 4.1. Patient and Carer Group Involvement

Coproduction is a term that refers to a way of working whereby decision-makers and service providers and users work together to create a decision or a service which works for them all [13]. The consultant nurses in each hospital approached members of their existing patient participation groups (such as those already involved in patient education) and invited them to take part in the PCG for this study. The PCG members were an integral part of the research team and participated in the research design, ethical approval process, design of the interview questions, undertaking of the interviews, data analysis, and dissemination of findings.

Members of the PCG had a role description and were reimbursed for their involvement, according to University and INVOLVE [14] guidance. Members of the PCG also underwent training for volunteers in each hospital and held a volunteer's contract which included a confidentiality agreement. They were supported by a renal counsellor and members of the research team throughout.

The training programme of three half-day sessions was delivered separately at each site over a four-week period. An outline of the training programme is shown in Table 1 and was delivered by both the university and hospital-based researchers.

### 4.2. Recruitment of Participants

In early 2015, participants were recruited by invitation letter. The inclusion criteria were those who were 70 years of age or older and who had commenced dialysis or made a decision not to dialyse within the previous six months. Those who were acutely ill and those who did not have the capacity to make a decision were excluded.

### 4.3. Interviews

One-to-one semistructured interviews with participants were undertaken. Semistructured interviews were chosen as the most appropriate method, as the alternative, which is structured interviews, can force respondents to choose from answers already provided and there is little opportunity for free expression [15]. In addition, semistructured interviews can allow the interviewer to focus on issues that are of particular importance to the research question, to probe and clarify comments made by the informant and to use prior knowledge to help him or her in this process [16].

Semistructured interview questions were originally devised from the literature review and based on a shared decision-making model as suggested by Stacey et al. [17]. Later, during the training sessions, the questions and prompts were more fully developed by the PCG (see Table 2), to enable the interviewers to encourage the participants to tell their story, as if in informal conversation.

Interviews were undertaken in the local hospital, usually in a private clinic room, and were scheduled according to patient preference (e.g., on dialysis days). There was provision made for interviewees who preferred to converse in their native language (e.g., Bengali), to have their interviews conducted by a bilingual member of the PCG and then translated into English. A renal counsellor was available for pastoral support of interviewees if required.

Participant identifiable data were only accessed by those in the direct health care team. Once participants were recruited, they were allocated a participant number (P1, P2, etc.).

Data on age, gender, ethnicity, and type of therapy (haemodialysis, peritoneal dialysis, or supportive care) were recorded but anonymised.

### 4.4. Analysis

Interviews were recorded using a digital recorder and transcribed by an external transcriber. Interview data were analysed by clinical members of the research team and the PCG together, using thematic data analysis. This is a conventional practice in qualitative research which involves searching through data to identify any recurrent patterns [18, p. 205]. Themes are a cluster of linked categories that convey similar meanings and usually emerge through an inductive analytic process, undertaken by the person(s) who has conducted the interview. Themes were identified using the technique of thematic analysis [19]. See “*Thematic Analysis (adapted from [19]*)” below. The researchers and PCG members were provided with anonymised transcripts of the interviews. These transcripts were read by each researcher/PCG member separately (Stage 1) and codes were generated (Stage 2). A PCG meeting then facilitated Stages 3 and 4.  Stage 5 was carried out by electronic communication. 

*Thematic Analysis (adapted from [19])*

**Stage 1 .**

Becoming familiar with the data.

**Stage 2 .**

Generating initial codes.

**Stage 3 .**

Searching for themes.

**Stage 4 .**

Reviewing themes.

**Stage 5 .**

Defining and naming themes.

### 4.5. Evaluation of Service User Involvement in the Project

Additionally, there needed to be a greater understanding on exactly what differences a PCG can make to the overall research process, especially implementation. A recent review has provided evidence of a range of benefits to researchers and participants of public and patient involvement (PPI) in health research [20]. We undertook an informal evaluation of the impact of the PCG on the research process (adapted from [20]) but explored how far PCG members felt valued as equal members of the research team and how far there was a measurable impact on the research design and delivery. A renal counsellor facilitated the evaluation of the focus group using some key questions to provoke discussion.

## 5. Results

### 5.1. Sample

In total 22 men and 7 women were interviewed, with a mean age of 77.4 years. One participant had an unplanned start to dialysis whilst the remainder had been referred in a timely way to nephrology services. 18 had chosen haemodialysis (HD), 6 had chosen peritoneal dialysis (PD), and 5 had chosen supportive care.

There were challenges in recruiting people who were representative of the diverse patient population in one hospital. Although more than 50% of adult incident population for RRT in one hospital is non-white [21], only 4/14 (28.5%) participants were non-white.

### 5.2. Themes

Overall the majority of participants felt that the medical and nursing staff were professional and caring and provided a range of comprehensive information sources (such as education sessions, written material, and DVDs) to aid in the decision-making process. There was much positive feedback from attendance at the regular seminars/patient days to enable questions to be asked face-to-face. The majority had the view that the dialysis decision was shared and that there was no undue influence from the medical or nursing staff.

Three main themes were identified following data analysis. These were* Delaying the Decision*,* The Decision-Making Continuum,* and* The Reality of Dialysis*.

### 5.3. Delaying the Decision to Start Dialysis

Many participants did not want too much information about dialysis too early in the decision-making process. In addition, if a decision to start dialysis did not need to be made within the forthcoming couple of months, then a number of participants said they would prefer to delay the decision until nearer the time.

One participant said

*He wanted to put me on dialysis for about nine months before I eventually went on because I kept putting him off, because we're great holiday people and we like to go on holiday and everything and I was trying to put him off all the time. (P1)*

Other participants realised that they were putting the decision off because they were anxious about dialysis and in denial of their symptoms.

*Yes, again I had the opportunity to go into the dialysis room and I thought, no, I'm putting that off tonight. It's like the execution day isn't it? (P5)*

Many participants spoke of feeling very scared about dialysis, especially following education input, and this may be one reason for putting off the decision to start replacement therapy.

*When I saw what happens on this big machine, wow, I had the fright of my life.” and “I saw this big thing on his hand (the fistula), and got the fright of my life. (P26)*

### 5.4. The Decision-Making Continuum

The continuum of shared decision-making was demonstrated by the majority saying they had been involved in the decision, yet one or two saying that they trusted the doctor to make the decision for them. Many people explained how the dialysis decision was truly shared, often between the individual and the health care professional (HCP). Participants felt that they had the opportunity to ask questions which were answered clearly and were also able to include members of their family/carers in the discussions.

When interviewees were asked whether their questions were answered, one participant said the following:

*They tell you to ask questions, they say is there anything you want to know, but you don't know the right questions to ask because you have no idea what the hell you're getting into. (P16).*

A number of participants explained the importance of having a family member with them, when dialysis options were being explained.

*They (family members) come with me. When I come here, they bring me…and they are educated people - they understand better than I do. (P29)*

We asked the participants if they felt their family influenced their decision; the response varied with one person saying

*Well I've only got one daughter and she's a nurse anyway, you know, she gave me advice on it with regards to was it worth it or such, and her verdict on it was that, yes, go for it. (P1)*

Another participant explained that family members did not influence their decision.

*I've got two daughters and a son and I spoke to them. His wife said to me, (because she's a little bit on the bossy side), you've got to have dialysis and I said why and she said you've got to go down that road and I said no. I said if I was younger I wouldn't think twice about it. (P2)*

However, some felt that although there were discussions they did not always understand what the doctor said and just trusted them to guide the decision. A smaller number described a process whereby the HCP had influenced the decision, with age being a particular theme in the context of the decision about whether to have dialysis or not. One said

*I did ask questions, and I said to him ‘The thing is, you tell me my age is against me', he said ‘Yes, it is against you', but he said it's entirely up to me, so I decided not to have it (dialysis). (P2)*

Some participants found the information provided about dialysis was too complex and was not easily understood. Several felt that although the information was good it was difficult to take it all in.

*I suspect that I had all the information at the time. What I didn't have was understanding. (P23)*

Others said there was too much information, especially the information gleaned from casual discussions in the waiting areas. One participant commented

*…so it's a waste of time listening to them or talking to them about it, because a lot of things I thought it's not as bad as it was. They all come here…and I thought I wish you hadn't said that because I've got to sit here listening to this, am I going to be alright, so the more information you've got is not really helping you at all. (P7)*

However, another found this type of casual information useful.

*When I was waiting to see the specialist, the ones that had come for dialysis, I listened to their conversations, and you can learn a lot that way. (P11)*

Others commented that peer support would not be useful.

*The only thing mentioned in that line is that there was a Patient's Association or some such name like that, that you could join and talk to other people in the same situation as you basically. Well I'm afraid I'm not one to join these sorts of clubs because I don't want to be bored with their hard luck stories and even more than that, I don't want to bore them with my hard luck stories. They are my problems, nobody else's problems and vice versa. (P6)*

### 5.5. The Reality of Dialysis

Although the availability of information was generally thought to be comprehensive, sometimes it appeared difficult to get answers about activities of daily living. One comment was that the one-to-one consultations and the group education sessions did not cover the bad points of dialysis. Particular reference was made to the need to hold large amounts of stock at home when undertaking peritoneal dialysis.

*Unfortunately I didn't realise there were other boxes as well with the tubes,…and of course it mean there were more boxes than I anticipated and as I say, it more or less took over the house. (P16)*

This participant also mentioned travel arrangements.

*Anyway I think the hardest part, the most aggravating part for me is the getting here, the parking, because I live in the middle of nowhere and I have to drive. (P16)*

The impact of dialysis was mentioned by a few participants to be understated.

*Both of them gave the impression that it was very easy, but nobody actually used the words life changing, and it is life changing. (P22)*

*Although they did glamorise it (the dialysis) a bit, they give the impression that you could sit and crochet for the four hours. (P22)*

A few participants asked for a single point of contact whilst on their kidney journey.

*I think if there was a bit more continuity, and I don't mean a personal relationship, but a best personal treatment where you could get the same nurse or staff nurse or sister on a more regular basis, because each time you go in you never know who you're going to get and they all seem to perform the function slightly differently from each other. (P17)*

And

*I think the only thing is you end up seeing so many different people, so there isn't anyone that actually controls your own situation, apart from yourself I suppose. So it might be helpful to have some sort of a managing agent effect, if you like, who's dealing with your treatment…. (P18)*

In summary, the participants in this study generally felt that the decision was shared and valued their family's involvement in the decision. However, at times, the information that was required to make the decision was sometimes provided too early and on occasion did not reflect the reality of dialysis.

### 5.6. Focus Group with PCG Members

The aim of the focus group was to evaluate, in a qualitative way, the impact that the PCG had on the research process [22]. Questions were developed by the research team following a review by Staley [22], and questions focused specifically on the impact of PCG involvement on the research delivery and the impact on the participants. The focus group was facilitated by a renal counsellor, who was part of the research team but not directly involved in the study. The focus group lasted for one hour and all members of the PCG (*n* = 6) were present. The focus group was recorded and thematic analysis was undertaken. Three main themes that arose from the focus group were* Experience of Interviewing*;* Personal Gain;* and* Feeling Valued.*

### 5.7. Experience of Interviewing

For the majority of the PCG members, this was the first type of research project they had been involved in, although two had acquired interviewing skills from previous jobs.

*I'd interviewed all my career, but this is a completely different type of interview. …As the interviews progressed I felt I was able to get more information out of people because I became more comfortable with the whole process.*

And

*What was different in this whole experience was asking people about their emotional experience …for me, finding the right questions and actually listening to myself making those questions was quite important because you don't want to come across at interrogatory, you want to come across as caring.*

Another member of the PCG spoke of how it was challenging to make the interviewees feel comfortable and trying not to pry into their personal lives. Another found it difficult to deal with emotional responses and said

*There was one person who started crying and it's a tough deal and you have to realise that people have had a life changing experience and listening to people saying I've just had enough of all this it's really quite tough. I came away feeling quite sad from a couple of the interviews.*

Overall the PCG feedback on the interview process was very positive and all members felt they had been as well prepared as they could have been. They learned a lot about themselves in the process and it helped them understand about listening and how to ask questions to this particular client group.

### 5.8. Personal Gain

Another theme that arose from the focus group was the variety of positive outcomes that arose serendipitously during the study. One member of the PCG said

*I was quite inspired by some people because you think when you're going through something yourself you suddenly realise that personally all I've got is kidney failure, I haven't got diabetes, I haven't got cancer or other organ failure. I found them just so positive and quite inspiring just to think my God, these people are making this decision not to have treatment at all and they seemed to be incredible people.*

Another said

*I thoroughly enjoyed it and glad I've been involved and hopefully the patients will have got something out of it, I certainly have and if nothing else met other people.*

### 5.9. Feeling Valued

The feedback from the focus group highlighted how much the members had benefited from being able to participate in a study which they could contribute to, rather than just being a participant. They felt they were listened to by the healthcare professionals, and their opinion mattered. They had an equal voice and one member of the PCG said

*We felt part of the whole project from the very beginning, although at times we found it hard to understand some of the processes such as ethical approval and the volunteer training.*

The PCG members talked about the advantages and disadvantages of involving patients in research, including an immediate camaraderie between interviewer and interviewee.

*It helped because they felt that you understood what they were telling you, there's almost that immediate bond.*

There were some issues with illness amongst PCG members. This did not delay the study, although it meant that one interviewer only undertook one interview.

## 6. Discussion

In general terms the findings suggested that participants were happy with the dialysis decision they had made and had been involved in the decision with their family and HCPs. This contrasts with previous studies that suggest that older patients feel insufficiently involved in the treatment choice [23].

### 6.1. Comparison with Other Study Findings

There are few previously published papers with a similar research question with which to compare findings. Harwood & Clark [24] suggested that dialysis modality decision-making processes for the older adult are very similar to how younger adults with CKD make modality decisions. A survey study in the USA [23] found that a significantly higher percentage of older patients felt the dialysis decision was made by the doctor rather than on their own or with their family or collaboratively with the doctor. In Harwood and Clark's [25] study, gender differences were noted between men and women. Compared with the women, the men in that study were less likely to seek out information regarding dialysis and more likely to delay making modality decisions.

Our study findings suggest that decision-making processes in our older population appear no different from a generic population, with few participants wanting a physician- or nurse-directed decision. Gender differences were not apparent, although only seven women were interviewed.

### 6.2. Impact on Research Design and Delivery

This is the first time a study on shared decision-making has been completely designed with patients and patients/carers being directly involved in interviewing patients who are from similar backgrounds. The PCG were able to empathise with the participants. There were occasions when information was provided by the interviewees about their dialysis experiences which (it was felt) may not otherwise have been forthcoming if a HCP had been interviewing them, in particular, with regard to the interviewees' personal and sometimes difficult decisions they made. The PGC felt that their relationship with the interviewees significantly reduced barriers that might otherwise have not been overcome. We also felt that it helped to put the interviewees at their ease when recalling what must have been a very difficult time in their lives.

Some PCG members did not undertake as many interviews as originally planned, with one interviewer undertaking just one interview because of illness. This did not impact our study as we had other interviewers who could step in; however this could potentially impact the trustworthiness of the data [26] if there is only one other interviewer available. It is important for coproduced projects to have processes in place for when coinvestigators are unwell. However, solutions to this potential issue do not appear to be well-reported, as no identified studies on user involvement in research appeared to discuss this in detail.

There were some issues with recruitment of participants from different ethnic groups, and, on reflection, these issues might have been ameliorated if the PCG members had been able to recruit participants directly. However, as Brett et al. [27] identified from a systematic review of 66 studies involving patients and the public, much of the evidence base concerning impact of patients and carers on research remains weak and needs significant work over the next ten years.

### 6.3. Limitations of Study

Rigour was maintained in the design and conduct of this study as far as possible using verification methods to ensure reliability and validity [28]. As with any novice interviewer, members of the PCG were supported by the research team and feedback given on the early interviews concerning interview technique and depth of probing.

An additional limitation was the recruitment difficulty in the inner-city trust with ethnic representation. This issue has been reported before [29] with individuals from minority ethnic backgrounds remaining frequently underrepresented in clinical studies. As these authors suggest, the forming of trusting relationships is pivotal to the successful recruitment of minority ethnic groups into research, and if a similar study is funded in future, members of the PCG would be involved in recruitment of participants.

### 6.4. Recommendations for Practice

The themes from the interviews suggest that healthcare professionals should consider tailoring information and patient education sessions to meet the needs of older people to support them in the decision-making process regarding renal replacement therapy. As Williams [30] suggests, there are many specific questions for older people who are making a dialysis decision: predictions about survival, quality of life, burden of the therapy, and the amount of recovery expected that must be answered by HCPs. At the same time, it must not be assumed that simply sharing this information equates to shared decision-making.

One specific way in which the findings of this study could inform clinical practice is involving peer supporters in disseminating the findings. Peer supporters are more widely being used in UK renal units to support those who are starting on their dialysis journey. One way in which peer supporters could help patients in their dialysis-decision is to provide real-life stories of others who have been through the decision, especially those who have delayed starting dialysis. For example, this quote “*when I walk out of here (the dialysis unit), I feel like a youngster again*” could be used to illustrate how much better people feel once they start renal replacement.

### 6.5. Recommendations for Future Research

It is recommended to repeat the study with younger patients and compare the findings with those from different age groups. In addition, it is important to measure the impact that patients and carers have on the research process. As Brett et al. [27] suggested that few studies have attempted any quantitative measurement of impact of patient involvement, reflecting the lack of robust tools available.

## 7. Conclusion

This coproduced study has highlighted the benefits of involving patients and carers in the design and process of qualitative research. It has enriched the findings of the study and enabled us to have a greater insight into the information needs of older people approaching dialysis. The involvement of patients as coresearchers has been a significant factor in its success. There has been a strong sense of ownership and responsibility to make it work and this is reflected in the comments from the PCG.

## Figures and Tables

**Table 1:** Patient and Carer Group training sessions.

Learning Outcomes	Content
(i) To understand the role of the Advisory Group members in undertaking interviews	Aims of project and rationale for method (group work)
(ii) To be aware of the knowledge, skills, and attitudes required to be a competent interviewer	What makes a good interviewer (brainstorm)
(iii) To be confident in undertaking an interview alone	Peer review of interviewing skills (role play)
(iv) To identify the practical issues involved in interviewing	Practicalities of interviewing (group discussion)
(v) To be competent and feel confident to carry out one-to-one interviews with support	Preparation for interviews: individualised learning needs (one-to-one support)

**Table 2:** Interview questions.

Theory of SDM (adapted from [17])	Interview questions
Recognition of the decision	Can you remember when you were told about having dialysis or not? Can you remember how you felt?
Knowledge transfer and exchange	Were you given any information? How far did you understand all the information you were given?
Expression of values and preferences	How much input did the doctors and/or nurses have in making the decision? How far do you think the decision was shared between you and your family?
Deliberation	Were you given an opportunity to think about the information? Was anyone with you to help explain the information? Did you try and find out anything more yourself?
Implementation of the decision	Did you feel supported in making your choice? Did you feel your own views were taken into account? Do you feel you made the right decision? Do you have any suggestions for improving the process?

## References

[1] Right Care http://sdm.rightcare.nhs.uk/pda/established-kidney-failure/.

[2] Murray M. A., Brunier G., Chung J. O. (2009). A systematic review of factors influencing decision-making in adults living with chronic kidney disease. *Patient Education and Counseling*.

[3] Kings Fund http://www.kingsfund.org.uk/sites/files/kf/Making-shared-decision-making-a-reality-paper-Angela-Coulter-Alf-Collins-July-2011_0.pdf.

[4] Coulter A., Ellins J. (2007). Effectiveness of strategies for informing, educating, and involving patients. *British Medical Journal*.

[5] Edwards A., Elwyn G. (2006). Inside the black box of shared decision making: distinguishing between the process of involvement and who makes the decision. *Health Expectations*.

[6] Moustakas J., Bennett P. N., Nicholson J., Tranter S. (2012). The needs of older people with advanced chronic kidney disease choosing supportive care: a review. *Renal Society of Australasia Journal*.

[7] Morton R. L., Tong A., Howard K., Snelling P., Webster A. C. (2010). The views of patients and carers in treatment decision making for chronic kidney disease: systematic review and thematic synthesis of qualitative studies. *British Medical Journal*.

[8] Levinson W., Kao A., Kuby A., Thisted R. A. (2005). Not all patients want to participate in decision making: a national study of public preferences. *Journal of General Internal Medicine*.

[9] Rosén P., Anell A., Hjortsberg C. (2001). Patient views on choice and participation in primary health care. *Health Policy*.

[10] Murray E., Pollack L., White M., Lo B. (2007). Clinical decision-making: patients' preferences and experiences. *Patient Education and Counseling*.

[11] Blackhall L. J., Murphy S. T., Frank G., Michel V., Azen S. (1995). Ethnicity and attitudes toward patient autonomy. *Journal of the American Medical Association*.

[12] Tariman J. D., Berry D. L., Cochrane B., Doorenbos A., Schepp K. G. (2012). Physician, patient, and contextual factors affecting treatment decisions in older adults with cancer and models of decision making: a literature review. *Oncology Nursing Forum*.

[13] Needham C., Carr S. (2009). *SCIE Research Briefing 31: Co-Production: An Emerging Evidence Base for Adult Social Care Transformation*.

[14] (2013). *Mental Health Research Network and INVOLVE, Budgeting for Involvement: Practical Advice on Budgeting for Actively Involving the Public in Research Studies*.

[15] Newell R. (1994). The structured interview. *Nurse Researcher*.

[16] Earnley C. (2005). A reflection on the use of semi-structured interviews. *Nurse Researcher*.

[17] Stacey D., Légaré F., Pouliot S., Kryworuchko J., Dunn S. (2010). Shared decision making models to inform an interprofessional perspective on decision making: a theory analysis. *Patient Education and Counseling*.

[18] Holloway I., Wheeler S. (2009). *Qualitative Research in Nursing and Health Care*.

[19] Braun V., Clarke V. (2006). Using thematic analysis in psychology. *Qualitative Research in Psychology*.

[20] Staley K. (2009). *Exploring Impact: Public Involvement in NHS, Public Health and Social Care Research*.

[21] Gilg J., Caskey F., Fogarty D. (2016). UK renal registry 18th annual report: chapter 1 UK renal replacement therapy incidence in 2014: national and centre-specific analyses. *Nephron*.

[22] Staley K. (2015). ‘Is it worth doing? Measuring the impact of patient and public involvement in research. *Research Involvement and Engagement*.

[23] Song M.-K., Ward S. E. (2014). The extent of informed decision-making about starting dialysis: does patients’ age matter?. *Journal of Nephrology*.

[24] Harwood L., Clark A. M. (2013). Understanding pre-dialysis modality decision-making: a meta-synthesis of qualitative studies. *International Journal of Nursing Studies*.

[25] Harwood L., Clark A. M. (2014). Dialysis modality decision-making for older adults with chronic kidney disease. *Journal of Clinical Nursing*.

[26] Noble H., Smith J. (2015). Issues of validity and reliability in qualitative research. *Evidence-Based Nursing*.

[27] Brett J., Staniszewska S., Mockford C. (2014). Mapping the impact of patient and public involvement on health and social care research: a systematic review. *Health Expectations*.

[28] Darawsheh W. (2014). Reflexivity in research: promoting rigour, reliability and validity in qualitative research. *International Journal of Therapy & Rehabilitation*.

[29] Rooney L. K., Bhopal R., Halani L. (2011). Promoting recruitment of minority ethnic groups into research: qualitative study exploring the views of South Asian people with asthma. *Journal of Public Health*.

[30] Williams A. W. (2014). Older adults with CKD and acute kidney failure: do we know enough for critical shared decision making?. *Journal of the American Society of Nephrology*.

